# Number of doses of Measles-Mumps-Rubella vaccine applied in Brazil before and during the COVID-19 pandemic

**DOI:** 10.1186/s12879-021-06927-6

**Published:** 2021-12-09

**Authors:** Tércia Moreira Ribeiro da Silva, Ana Carolina Micheletti Gomide Nogueira de Sá, Ed Wilson Rodrigues Vieira, Elton Junio Sady Prates, Mark Anthony Beinner, Fernanda Penido Matozinhos

**Affiliations:** 1 Department of Maternal and Child Nursing and Public Health, School of Nursing, Universidade Federal de Minas Gerais, Belo Horizonte, Minas Gerais 30190 000 Brazil; 2 Graduate Programa in Nursing, School of Nusing, Universidade Federal de Minas Gerais, Belo Horizonte, Brazil

**Keywords:** Measles-Mumps-Rubella vaccine, Epidemiology, COVID-19, Brazil, Ecological studies, Spatial analysis

## Abstract

**Background:**

Due to the social isolation measures adopted in an attempt to mitigate the risk of transmission of SARS-CoV-2, there has been a reduction in vaccination coverage of children and adolescents in several countries and regions of the world.

**Objective:**

Analyze the number of doses of vaccine against Measles-Mumps-Rubella (MMR) applied before and after the beginning of mitigation measures due to COVID-19 pandemic in Brazil.

**Methods:**

The data collected refer to the number of doses of the MMR vaccine applied monthly to the target population residing in Brazil: cahildren, aged 12 months (first dose) and children, aged 9 years (second dose), from April 2019 to December 2020. Differences in MMR vaccine doses from April 2019 to March 2020 (before the start of mitigation measures) and April 2020 to September 2020 (after the start of the mitigation measures) were evaluated. Spatial analysis identified clusters with a high percentage of reduction in the median of applied doses no Brazil.

**Results:**

There was a reduction in the median of doses applied in the Regions North (− 33.03%), Northeast (− 43.49%) and South (− 39.01%) e nos Estados Acre (− 48.46%), Amazonas (− 28.96%), Roraima (− 61.91%), Paraíba (− 41.58%), Sergipe (− 47.52%), Rio de Janeiro (-59.31%) and Santa Catarina (− 49.32) (p < 0.05). High-high type spatial clusters (reduction between 34.00 and 90.00%) were formed in the five regions of Brazil (Moran’s I = 0.055; p = 0.01).

**Conclusion:**

A reduction in the number of MMR vaccine doses was evidenced as a possible effect by the restrictive actions of COVID-19 in Brazil.

## Background

During the COVID-19 pandemic, national and international health agencies recommended that immunization services to continue, uninterrupted, their activities, due to the possibility of a return of vaccine-preventable diseases, controlled or eliminated, in response to low vaccination coverage [1]. However, studies have shown that, due to the social isolation measures adopted in an attempt to mitigate the risk of transmission of SARS-CoV-2, the etiological agent of COVID-19 [2, 3], there has been a reduction in vaccination coverage of children and adolescents in several countries and regions of the world [4, 5].

The impact on vaccine coverage was not an exclusive event of the COVID-19 pandemic. Studies conducted after catastrophes and epidemics that occurred in human history also pointed to a decline in vaccination coverage as a response to the reduction in the population’s supply and access to health services [6].

Difficult access to immunization services and child malnutrition are factors that act synergistically, placing communities living in a situation of social vulnerability more susceptible to the development of severe forms and death as a result of measles [7]. Even with the widespread diffusion of the Measles-Mumps-Rubella vaccine (MMR vaccine) in 2019, the world's measles rates reached their highest level over the last two decades [7, 8]. In fact, it is estimated that in 2018, more than 140,000 people died from measles, with the majority of deaths reported in underdeveloped countries, affecting primarilarly malnourished children [8]. In this sense, health strategies and policies aimed at improving MMR vaccine coverage indicators are needed, especially in low- and middle-income countries [7].

The MMR vaccine, produced from live attenuated measles, rubella and mumps viruses, is available free of charge in Brazil and recommended for routine vaccination at 12 months and 9 years of age [9]. Even with the freely available vaccine distributed throughout the national territory, in 2018, two years after receiving measles-free certification for the elimination of virus in the Americas [10], 10,346 cases of the disease were confirmed in Brazil, resulting in the loss of certification as a “measles virus-free country” [11]. The recently reported cases of measles can be explained by the progressive drop in the coverage of the MMR vaccine in Brazil over the last decade and by the formation of clusters of susceptible individuals in the States of Acre, Amazonas, Pará, Amapá, located in the North region, and in the State of Maranhão, located in the Northeast region of the country [12].

Considering that the historical reduction in MMR vaccine coverage rates in Brazil [13, 14] may have been compromised by the sanitary measures adopted due to the COVID-19 pandemic and that in Brazil the distribution of health services and the allocation of health resources are heterogeneous [15], this study aimed to analyze the number of doses of the MMR vaccine applied before and after the beginning of social distancing measures in response to the COVID-19 pandemic that took effect in the municipalities, states and regions of Brazil. Brazil ranked third among the countries with the highest number of confirmed cases of COVID-19 (more than 22 million on September 30, 2020, according to the WHO) [16], in addition to the absence of a comprehensive national plan to combat the pandemic and to adequately secure the reduction of mortality from the highly contagious disease [17]. In addition, this study also aimed to identify, through spatial analysis, clusters formed by municipalities with a high contingent of individuals susceptible to measles.

## Methods

### Study design

This was an ecological study, with data taken from the Brazilian National Immunization Information Program System (SI-PNI), available at http://sipni.datasus.gov.br/. The SI-PNI provides the number of monthly doses of vaccines applied throughout the country.

### Data collection

The data collected refer to the number of doses of the MMR vaccine administered in the period from April 2019 to September 2020. Data extraction was performed by the number of monthly doses applied to the target population over the period: children, aged 12 months (first dose) and 9-year-old children (second dose).

### Variables

The independent variable was the number of doses applied. The independent variables were geographical, including the five regions of the country (North, Northeast, Central, Southeast and South), the 27 States of the Federation, which are comprised of 5568 Brazilian municipalities.

### Statistical analysis

First, the doses of the MMR vaccine applied before (April 2019–March 2020) and after the beginning of social distancing measures in Brazil (April–September 2020) in the 27 states were added. Next, the differences between the median number of doses applied before and after social distancing measures were evaluated using the Mann–Whitney U test and the significance level was established at 5%. The percentage of variation of the median doses applied was estimated using the following equation:

$$\left[ {\left( {{\text{median of doses applied by States before social distancing measures}} - {\text{median doses applied by State after social distancing measures}}} \right) \, /{\text{ median of doses applied by States before social distancing measures }} \times { 1}00} \right].$$

These analyzes were processed using the Statistical Package for Social Sciences software (IBM-SPSS, v.19, IBM, Chicago, IL).

For the general spatial analysis, the percentage variation of the median doses of the MMR vaccine was considered before and after the beginning of social distancing measures in Brazil for each Brazilian municipality. The percentage variation of the median of applied doses was estimated using the equation previously mentioned.

Techniques for spatial analysis of area data were used considering the digital grids of the Brazilian municipalities, using two Geographic Information System (GIS) programs. To examine the existence of a spatial correlation of the median reduction of doses of the MMR-Triple Viral vaccine, the Global Moran’s Index (I) was calculated, which ranges from − 1 to + 1, with positive values (between 0 and + 1) indicating direct correlation and negative values, between 0 and − 1 (an inverse correlation). Spatial correlation is interpreted according to the I and can translate to weak (I < 0.3), moderate (I ≥ 0.3; < 0.7) or strong (I > 0.7) [18].

From the cartographic base of the Brazilian municipalities acquired on the IBGE website, cartograms were created for the presentation of clusters with statistical significance (p < 0.05). The Moran Eigenvector Maps (MEM) show the high-high spatial clusters (red color) resulting in statistical significance, formed by municipalities with a high percentage reduction in the median of applied doses of the MMR vaccine and surrounded by municipalities with the same trend. Municipalities that failed to present significant spatial correlation (p > 0.05) or that formed low-low, low–high or high-low type spatial clusters, were excluded from the map. The regions of the country, namely: North, Northeast, Central, Southeast and South, are represented on the cartogram with different shades of gray.

In this study, the 95% of Global Moran I level of significance was considered after 999 permutations [18], that is to say, the areas with statistically significant spatial correlation were those whose p-value was less than or equal to 0.05 after 999 random permutations. For these spatial analyses, the following software was used: Spatial Analysis Laboratory, University of Illinois, Urbana Champaign, United States (GeoDa 0.9.9.10) and TerraView, version 4.1.0.

### Ethical aspects

Due to the nature of this study of using freely accessible data, it was not necessary to submit the present study to the Research Ethics Committee, in accordance with Resolution 466/2012 of the National Brazilian Health Council [19].

## Results

From April 2019 to September 2020, 25,717,742 doses of the MMR vaccine were applied throughout Brazil (46.55% at beginning of the social distancing measures). In the period before measurements, the median number of doses applied was 1,645,527. During this period, the median dropped to 934,991, equivalent to a reduction of 43.17%.

Of the five regions in the country, the North, Northeast and South showed a statistically significant reduction in the median number of doses applied while the the public health emergency measures were in place. Among the states, seven demonstrated a statistically significant reduction, ranging from 47.52% in Sergipe, to 64.91% in Roraima (Table 1).

**Table 1:** Median and percentage change in the median number of Triple Viral vaccines (MMR) administered

States and Regions	Number of dose per eligible population	April/19–March/20 Median	April/20–September/20 Median	Change* (%)	p**
Brazil	2,923,441	1,645,527	934,991	− 43.17	0.18
North			5,524	− 33.03	0.01
Acre	16,358	3,725	1,920	− 48.46	0.01
Amapá	15,399	4,210	1,811	− 56.97	0.07
Amazonas	78,049	24,865	17,662	− 28.96	0.00
Pará	138,682	7,060	47,808	− 32.31	0.68
Rondônia	27,503	9,853	7,309	− 25.81	0.25
Roraima	11,737	8,164	2,864	− 64.91	0.00
Tocantins	24,932	6,519	5,471	− 16.07	0.18
Northeast			17,618	− 43.49	0.01
Alagoas	50,368	24,748	12,669	− 48.80	0.55
Bahia	204,086	72,075	62,320	− 13.53	0.49
Ceará	127,797	49,394	38,333	− 22.39	0.49
Maranhão	112,981	41,476	25,885	− 37.58	0.06
Paraíba	57,493	18,047	10,543	− 41.58	0.01
Pernambuco	135,906	45,644	41,979	− 8.03	0.55
Piauí	48,551	22,437	12,136	− 45.90	0.13
Rio Grande do Norte	46,222	14,910	10,454	− 29.89	0.44
Sergipe	33,867	14,318	7,513	− 47.52	0.04
Central			13,181	25.66	0.08
Distrito Federal	44,568	16,022	11,968	− 25.29	0.44
Goiás	97,515	26,599	22,884	− 13.96	0.55
Mato Grosso	57,268	12,770	12,194	− 25.84	0.18
Mato Grosso do Sul	44,747	12,769	12,193	− 4.51	0.68
Southeast			76,034	− 28.87	0.35
Espírito Santo	55,846	21,798	25,013	14.74	0.89
Minas Gerais	260,957	110,574	140,480	27.04	0.75
Rio Janeiro	223,216	89,087	36,248	− 59.31	0.02
São Paulo	611,798	246,595	226,283	− 8.23	0.34
South			33,084	− 39.01	0.01
Paraná	157,693	81,645	57,506	− 29.56	0.75
Santa Catarina	98,334	56,141	28,447	− 49.32	0.01
Rio Grande do Sul	141,568	43,931	30,522	− 30.52	0.15

P = Percentile; *Consists of the percentage change in the median number of Triple Viral vaccines (MMR) administered; − reduction; + increase; **Mann–Whitney test (difference between medians)

Source: National Immunization Program, Brazil, April 2019 to March 2020 and April 2020 to September 2020

**Fig. 1:**
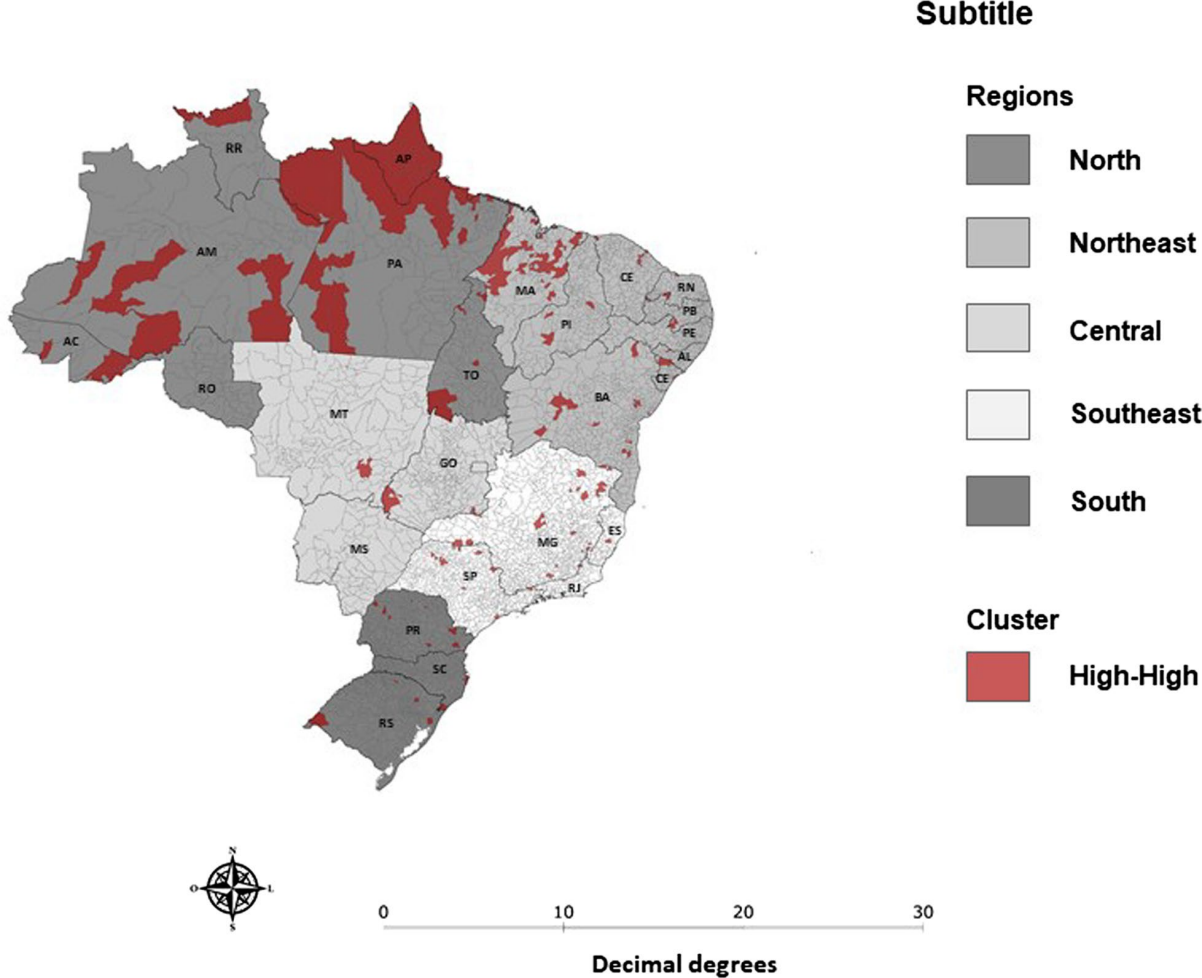
High-high spatial clusters with statistical significance Source: National Immunization Program, Brazil, April 2019 to March 2020 and April 2020 to September 2020

Weak spatial autocorrelation (I = 0.055; p = 0.01) and the presence of High-High spatial clusters were identified, formed by 262 municipalities that presented a reduction in the median of applied doses between 34 and 90%, 88 of which were located in the North Region, 107 in the Northeast region, 41 in the Southeast region, and 26 in the South region (Fig. 1).

## Discussion

The COVID-19 pandemic resulted in a reduction in the number of applied doses of the MMR vaccine as a possible effect of the restrictive actions of COVID-19. The North, Northeast and South regions and the States of Acre, Amazona, Roraima, Paraiba, Sergipe, Rio de Janeiro, and Santa Catarina showed a significant reduction in the median of MMR vaccine doses applied during the period that recommendations for social distancing were instituted in Brazil. High-High spatial clusters were formed by municipalities located mostly in the Northeast and North regions of the country.

National and international studies attributed a reduction of the population’s demand for health services, with a consequent drop in vaccination coverage, due to the restrictive mitigation measures adopted during the COVID-19 pandemic [1, 5, 20, 21]. However, there has been an observed trend in a decline in vaccine doses applied in Brazil over the last two decades [21], especially those immunobiologicals recommended during early childhood [12, 22]. Contextual and individual factors, cited in recent studies [21, 23], have attributed the decline based on vaccination coverage including a lack of planning by the Brazilian National Universal Healthcare System (SUS), social and cultural aspects effecting vaccination acceptance, logistical difficulties cited by the PNI in offering several routine vaccines as part of the national vaccine schedule, anti-vaccination movements, and inconsistencies in the availability of immunobiologicals offered by Primary Healthcare services [24–27].

The results of the present study demonstrate that the number of MMR vaccine doses applied between April 2020 and September 2020 was considerably lower than in the period from April 2019 to March 2020. This result does not rule out evidence that contextual factors and individuals have acted synergistically, contributing to the reduction in the number of applied MMR vaccine doses [12, 25, 27, 28], but highlights that social isolation, triggered by the COVID-19 pandemic, greatly contributed to the reduction in the number of applied MMR vaccine doses. This fact was evidenced by the sudden change in the behavior of the absolute numbers between the evaluated periods and confirmed by statistical analyses.

Also in this study, three of the five Brazilian regions demonstrated a statistically significant reduction in the median of applied MMR vaccine doses during the period of social distancing measures. This scenario, added to the drop in vaccination coverage rates in recent years, point to a problem for collective immunity and the risk of outbreaks resulting from the measles virus [22, 29]. Furthermore, it is worth noting that the regional inequalities in vaccination coverage in Brazil has favored the formation of pockets of susceptible individuals [12, 22, 30].

Between 2015 and October 2018, Brazil experienced a significant drop in MMR vaccine coverage, from 96.1 to 86.7%, and only after the national vaccination campaign, in September 2018, did it reach the 95% target [13, 31]. These low vaccination coverage indicators, added to measles cases, imported from Venezuela, triggered an epidemic of the disease that affected several Brazilian states, mainly states in the Northern regions [32].

The findings of present study showed that, added to the historical reduction in the coverage of the MMR vaccine, as highlighted in the literature [33], there was a sharp drop in the number of doses of the MMR vaccine applied during the first six months, when compared to the previous period of the COVID-19 pandemic. A study that evaluated the availability of the MMR vaccine in Brazil, from 2013 to 2014, reported that the immunization services, located in the Northern region, possessed inadequate infrastructure for undertaking immunization actions which resulted in a lower frequency of vaccine availability [23]. The lack of vaccine in the Northern region, even during a short period of time, incurs a lost opportunity for vaccination and can compromise the achievement of vaccination coverage goals, increasing the number of susceptible individuals from that region [23].

The lower frequency of availability of the MMR vaccine, on top of the logistical and infrastructure problems of the primary healthcare services in the Northern region, may have contributed to the formation of clusters with a higher percentage reduction in the coverage of the MMR vaccine in this region. In this study, by using spatial analysis, we identified cities that showed a reduction in the number of applied MMR vaccine doses and that were close to neighboring cities demonstrating a similar behavior of forming a cluster. The concentration of individuals who are not adequately immunized with the MMR vaccine compromises collective immunity, increases the risk of circulating disease such as measles, rubella and mumps, and therefore, the identification of these areas is essential to fast track public health policies and health strategies for improvement of immunization indicators [33].

In the North and Northeast regions of the country, we identified a greater number of clusters that showed a reduction in the number of applied MMR vaccine doses. Regional inequalities in vaccine coverage in Brazil [12, 29] can be attributed, in part, to differences in investments in the health sector in the North and Northeast regions when compared to other Brazilian regions, which culminated in the precariousness of the nationally mandated Primary Healthcare Services (*Atenção Primaria*—AB), responsible for offering free immunization through the National Immunization Program (PNI) [23].

Furthermore, it is noteworthy that measles is one of the most contagious infectious diseases known [34], requiring the adoption of emergency strategy actions for vaccinating communities that formed clusters with a significant reduction in immunization coverage during the COVID-19 pandemic period. This strategy aims to reduce the chances of overlapping cases of measles and COVID-19, which could favor the collapse of healthcare services in these regions.

The collapse of health services in some states in the North and Northeast regions, due to the increasing demand for hospital beds for patients with COVID-19, may have contributed to the reduction in the population's demand for immunization services in these regions [35, 36], resulting in the formation of clusters with higher percentages of reduction in the applied MMR vaccine doses in these regions.

Strategies to contain the pandemic in states and regions of Brazil were also not uniform, which may explain the percentage variations in the median of applied MMR vaccine doses, from 47.52%, in the State of Sergipe (p = 0.04), to 64.91% in the State of Roraima (p = 0.00). While in some locations, the response to the epidemic phase of acceleration of the number of reported cases and deaths from COVID-19 was using mitigation practices through social distancing. Other locations resorted to the strategy of total confinement, that is, suspending all non-essential activities and limiting the circulation of people [37, 38].

In Brazil, more than a year after the first case of COVID-19, the country continues to lag behind many developing countries in an effort to immunize its population against COVID-19 [39] and many public health officials agree that long-term social isolation strategies will continue for several years to come [40]. Under this scenario, it is vital to adopt health strategies and policies that ensure the population's universal access to immunization programs. The consequences of a lack of access would mean living with the overlapping cases and deaths from COVID-19 with other infectious diseases, such as measles, rubella and mumps.

### Limitations and study strengths

One of the weaknesses of the present study was in relation to the intrinsic limitations of studies that use secondary data, in addition to the fact that the available data were not specifically collected to answer the questions proposed in this research. Another point that deserves to be highlighted was the possible influences related to the standardization and quality of filling in the SI-PNI records, which may be subject to information bias. The SI-PNI is considered a powerful tool as a source of data on immunizations for the Brazilian population, and even with some weaknesses, as pointed out, it has solid bases, with more than 40 years of existence, contributing to support monitoring and decision-making in the country [32, 33]. The SI-PNI is useful in the fulfillment of its mission, decisive both in the control and enabling the identification of groups susceptible to vaccine-preventable diseases through individualized data and the management of actions throughout the Brazilian territories [34]. A study that evaluated the SI-PNI revealed that it is a system that maintains sensitive criteria, managing to capture a satisfactory percentage of vaccination of the population [34]. In addition, the SI-PNI is coordinated by the Ministry of Health [32] and possesses strict regulations for control, adherence and standard fulfillment targets [34]. In this way, the data presented here are recognized and very close to the reality in Brazil. In this study, the SI-PNI registered population data was used during the study period, and the generalization of these results is relatively safe for national estimates. Also, to control biases, methodological rigor was taken into account during all of the stages of the study execution.

## Conclusions

The COVID-19 pandemic resulted in a reduction in the number of applied MMR vaccine doses as a possible effect from the restrictive actions against COVID-19. In Brazil, few studies have evaluated the impact of the COVID-19 pandemic on the vaccination of children and adolescents, and this is the first study in the country to consider the MMR vaccine. The results of this work may support public health policies to guarantee immunization strategies against measles, rubella and mumps in the country, even during the current epidemic phase, which continues to result in increased numbers of reported cases and deaths from COVID-19 in Brazil. In this sense, this work may highlight priority areas for which public health policies and health strategies should be adopted to improve immunization indicators, in order to prevent the spread of potentially vaccine-preventable infectious diseases.

## Data Availability

The datasets used and/or analysed during the present study are available from the corresponding author upon request.

## Abbreviations

COVID-19: Coronavirus Disease Pandemic
MMR vaccine: Measles-Mumps-Rubella vaccine
I: Global Moran’s Index
MEM: Moran Eigenvector Maps
PNI: National Immunization Program
IQR: Interquartile range
SI-PNI: Brazilian National Immunization Information Program System
